# Monitoring the infection rate: Explaining the meaning of metrics in pandemic news experiences

**DOI:** 10.1177/14648849221149599

**Published:** 2023-01-03

**Authors:** John Magnus R. Dahl, Brita Ytre-Arne

**Affiliations:** MediaFutures - Research Centre for Responsible Media Technology & Innovation, 1658 University of Bergen, Bergen, Norway; Department for Information Science and Media Studies, 1658 University of Bergen, Bergen, Norway

**Keywords:** COVID-19, news use, emotions, valuable journalism, data visualization, metrics

## Abstract

The COVID-19 pandemic has brought forward questions of what citizens need and want from journalism in a global crisis. In this article, we analyse one particular aspect of pandemic news experiences: Preoccupation with monitoring metrics for COVID-19 infection cases, hospitalisations, and deaths, widely disseminated through journalistic news outlets. We ask why close monitoring of such metrics appeared meaningful to news users, and what these experiences can tell us about the role of journalism in the pandemic information environment. Our analysis draws on qualitative research conducted in Norway in 2020, finding users particularly devoted to monitoring metrics, both in early lockdown and during the second wave of COVID-19. To contextualize our findings, we draw on scholarship on emotional responses to data in the everyday, and on the social role of journalism. We argue that monitoring of infection rates is an expression of trust in the media as a provider of factual information, also expressed by those who are cynical towards other aspects of journalism, and we conceptualise this monitoring practice as a coping strategy to deal with the pandemic as an unknown and uncontrollable threat, involving difficult emotions of uncertainty and fear.

## Introduction

Metrics are central to the ways the COVID-19 pandemic has been reported and were particularly central in the first year of pandemic journalism. Rising infection rates were interpreted as precursors of concern, detailing how the pandemic moved across the world and fluctuated through different waves. Case numbers have been supplemented with metrics for hospitalizations, deaths, and eventually vaccination coverage. Governments and health authorities have contributed to making metrics integral to public and strategic communication about the pandemic, although such uses can be interpreted as obfuscating as well as clarifying the situation (Billig, 2021; Dudziak, 2021; Lawson and Lugo-Ocando, 2022). Information about pandemic metrics can be found on tracking websites such as Worldometer, but journalistic news outlets have informed people of the latest numbers in their respective local or national communities, as well as reporting international developments. In 2020, such reports typically took the form of televised press conferences with local officials, publishing “the number of the day” in headlines and news feeds, or integrating data visualization tools in journalism on the pandemic situation.

In Norway, a highly digital news market (Newman, 2021), several newspapers amended their online news fronts through the pandemic to highlight metrics. This included the largest tabloid and most popular online newspaper, VG, which won widespread recognition and a journalistic award for a project for reporting and visualizing coronavirus metrics, including an interactive statistics banner at the top of the online news front (VG 3.5.2021). Regional newspapers owned by the same media company, Schibsted, also featured similar banners reporting on infections, hospitalizations, intensive care patients, deaths, and counter-pandemic measures, with several options for customization to local, national, or international locations. When a surprising plan for immediate lifting of all restrictions was announced by the prime minister in fall 2021, VG replaced the statistics banner with a clock counting down to online fireworks and confetti on the front page – but a few weeks later Omicron surged, and the banner returned.

In this article, we analyse the experience of monitoring COVID-19 infection metrics from the perspective of news users, asking why close monitoring of such numbers appeared meaningful, and what these experiences can tell us about the role of journalism in the pandemic. We have conducted a qualitative in-depth interview study with Norwegian news users in late 2020, asking people to recount their pandemic news experiences from first hearing of COVID until the time of the interview, which was characterized by second waves and new restrictions. A key finding was preoccupation with numbers, as people told us metrics constituted the most valuable information they could get from pandemic news. In this article we delve deeper into what this means, applying theories from data studies on everyday engagement with metrics (Kennedy and Hill, 2018; Radinsky and Tabak, 2022), and from journalism studies on expectations to journalism’s societal role (Costera Meijer and Bijleveld, 2016; Nielsen, 2017). We answer the following research questions: Why did monitoring of infection metrics appear meaningful to news users? What can these experiences tell us about everyday engagement with data and the role of journalism in the pandemic information environment?

We conceptualise monitoring practices as coping strategies to deal with the pandemic as an uncontrollable threat, involving difficult emotions of uncertainty and fear. Furthermore, we highlight expressions of trust in the media as a provider of factual information, also expressed by those who were cynical towards other aspects of journalism’s societal role. Thus, monitoring the infection rate constituted an information shortcut used for practical purposes of interpreting public health guidelines, and for emotional work, to translate a fuzzy object of fear – the pandemic – into something more specific and less scary. Our analysis contributes to the literature on pandemic news use, and more broadly to understandings of what is experienced as valuable journalism, with a specific example indicative of broader sense-making practices. This example is interpreted by making an explicit connection between the literature of everyday data practice and the literature on valuable journalism.

In the following, we first discuss theoretical perspectives on valuable journalism and the everyday engagement with data and metrics, before detailing the methods of our qualitative study. Our analysis focuses on monitoring as a coping strategy, and perceptions of numbers as particularly trustworthy and relevant, before we conclude by discussing implications.

## Valuable journalism in pandemic crisis

News use through the pandemic is a topic that has attracted considerable scholarly interest (Broersma and Swart, 2021; Mihelj et al., 2021; Newman, 2021; Van Aelst et al., 2021). Studies in different countries have found similar patterns of intensified news use in early 2020, but also documented experiences of information overload and news fatigue as the pandemic continued (Ytre-Arne and Moe, 2021; Nguyen, n.d.; De Bruin et al., 2021; Groot Kormelink and Klein Gunnewiek, 2021; Vandenplas et al., 2021; Mannell and Meese, 2022). The studies indicate partly different findings on the meaning of metrics in pandemic news: Groot Kormelink and Klein Gunnewiek (2021) quote young news uses who declared daily numbers were not important, but also find that modes of information-seeking and avoidance seemed to fluctuate with infection rates. Mannell and Meese (2022) found their informants using case numbers as a proxy for the lockdown situation overall, thus enabling selective monitoring of only the numbers of the day, as part of overall patterns of avoidance. Vandenplas et al. (2021) refer to numbers as key to news surveillance in early lockdown, but also as an example of emotionally draining content people might want to avoid. These findings, as well as the sheer extent of coronavirus news, point to needs for news users to prioritize in their engagements with the pandemic information flow, provided across journalistic media and other digital platforms. This situation also begs the question of what users want from journalism in a crisis such as the COVID-19 pandemic, and how particular types of news are experienced as valuable under these special circumstances.

The notion of “valuable journalism” has been developed by Irene Costera Meijer (Meijer, 2013; Costera Meijer and Bijleveld, 2016) to address user experiences in debates on quality news content. In this analytical framework, important dimensions of how users experience valuable journalism are participation, representation, and presentation. Instead of ascribing quality or value to certain types of news, the focus is on whether users experience that their concerns and interests are integrated into the journalistic process and product. A different study with a similar framework (Costera Meijer and Bijleveld, 2016) points to four dimensions – urgency, public connection, regional understanding and audience responsiveness – as important to experiences of value. While we can presume that a crisis like a global pandemic could affect perceptions of what constitutes valuable news, the dimensions appear relevant also in crisis – such as users experiencing news as applicable to their daily lives, in their communities. We argue that everyday engagements with metrics in the pandemic constitutes an example of valuable journalism in this regard.

The discussion on valuable journalism connects to debates on the societal role of the news media, in a fragmented high-choice media environment characterized by information abundance, where news is mixed into streams of information across digital platforms (Bengtsson and Johansson, 2020; Boczkowski, 2021). Citizens navigating such information environments have been described as approximately informed and occasionally monitorial (Ytre-Arne and Moe, 2018), and research into news use details various shortcuts and practices of checking in with the news (Ørmen, 2016; Meijer and Groot Kormelink, 2020). Rasmus Kleis Nielsen has argued, based on a democratic-realist approach, that the one thing journalism might do for democracy is to “provide people with relatively accurate, accessible, diverse, relevant, and timely independently produced information about public affairs” (Nielsen, 2017). This understanding highlights *information* as particular to what journalism (as opposed to other societal institutions) can specifically provide for democratic societies. Information provision is central also to understandings of journalism’s social role in everyday life: Hanitzsch and Vos have outlined a wide-reaching typology of different roles for journalism in political and everyday life (Hanitzsch and Vos, 2018), with mood management, guiding and service provision as keywords for the everyday sphere. This connects to the notion of “service journalism” informing and assisting people about everyday life decisions, including how to assess risks and give guidance in a chaotic world (Eide and Knight 1999; From and Kristensen 2018). In a pandemic, the potential tasks journalism might take on are vast, and even if information provision is considered one of many specific tasks, this appears in need of further operationalizations.

Metrics and their visualization appear as one possible operationalization of complex pandemic information. While numbers often are framed as inherently objective and thus as ‘pure’ information, they are also used in diverse ways both rhetorically by governments (Billig, 2021) and in everyday information practices (Kennedy and Hill, 2018; Radinsky and Tabak, 2022). Scholarship on data in the everyday is particularly relevant to understand how COVID-19-metrics were experienced as a particularly valuable form of journalism.

## The emotional everyday engagement with numbers

Scholarship on datafication has typically focused on the macro and meso levels of the social world, with Mayer-Schönberger and Cukier (2013) and Zuboff (2019) as examples that have gained significant attention even outside of academia. However, there has recently been increased interest in how social actors engage with data, including how individuals experience data in their everyday lives (Livingstone, 2019; Milan et al., 2020). A strand of this literature is concerned with how people relate to numbers and their visualisation, departing from an everyday life perspective with interest in how data are experienced from day-to-day and interwoven in the way people live (Kennedy and Engebretsen, 2020; Kennedy and Hill, 2018). A relevant example concerning how data visualisations are used for everyday monitoring is the weather forecast, which has been shown to play a significant role in the planning of everyday activities and for experiencing control in everyday life (Masson and Van Es, 2020; Sturken, 2001).

Interpreting data and data visualisations are thus part of everyday life in the form of data practices. In the context of the covid 19-pandemic, Radinsky and Tabak (2022) identify three such practices: *scanning, looking closer,* and *puzzling through;* as well as three dimensions relevant for understanding how these practices were used to make sense of everyday life in the pandemic: *agency, emotion,* and *trust.* While the three practices can be carried out independently of each other, the three dimensions are inherently intertwined, and show how the use of numbers in everyday sensemaking goes beyond a mere search for objective or accurate information, while still hinging on the belief that numbers can provide such information.

It should be emphasised that although studies drawing on cognitive psychology tend to argue that numbers in journalism elicit little emotional response (see for example Maier et al., 2017), data scholars departing from an everyday life perspective arrive at the opposite conclusion. Numbers can be used as a tool to regulate emotion, but also evoke strong emotional reactions: Through their ability to create distance and abstraction, numbers can appear as objective (Porter, 1996), but the very same capacity for abstraction allow them to be experienced as the ‘data sublime’ (Davies, 2015; Gray, 2020), where their “sheer quantitative magnitude” can elicit ambivalent feelings of awe. Both emotional reaction and emotional regulation are part of how individuals use data to make sense of the world and ultimately comport themselves as social actors (Kennedy and Engebretsen, 2020).

This emotional dimension of everyday engagement with COVID-19 metrics is, in our opinion, also key to understand how metrics are experienced as valuable journalism, as we will argue below. Although emotional aspects of journalism have received increasing attention (Wahl-Jorgensen and Pantti, 2021), the emotional component of *journalism as information* has largely been neglected. Earlier studies (Groot Kormelink and Gunnewiek, 2021) indicate how seeking information appears as a means of striving to regain stability in one’s views of the world in the emotionally charged pandemic new use. Our findings indicate that the monitoring of seemingly specific and factual numerical information on infection rates is an important part of this process. Understanding what this monitoring means to news users is therefore key to understand how metrics can be experienced as valuable journalism.

## Methods: qualitative in-depth interviews on pandemic news experiences

Our study builds on qualitative research on news users through the COVID-19 pandemic in Norway, with an interview study conducted during a second wave of infections in late fall 2020.

As the Norwegian context is central to our study, it is important to underline the significance of this geographical location as well as the point in time in the COVID-19 crisis. Norwegian society is marked by high levels of trust both in media and authorities (Syvertsen et al., 2014), which has been attributed as important to a relatively positive pandemic outcome, with high compliance with guidance and trust in information (Sætrevik, 2020, Knudsen et al., in press). Norway has a comparatively low death rate from coronavirus (Yarmol-Matusia et al., 2021). Like many other European countries, Norway experienced the first COVID cases in early 2020, and went into an unprecedented national lockdown in March. This lockdown was announced by prime minister Erna Solberg in a press conference on March 12^th^, a time of intense uncertainty about what the virus threat entailed, with discussions about whether infections had already spiralled out of control. However, the measures instituted in Norway appeared to take effect very soon, and the first lockdown was lifted as soon as a few weeks later, allowing schools to re-open before a summer of low infection rates and extensive optimism. In fall 2020, infections started rising again, predominantly in major cities, and in late fall 2020 the capital Oslo went into lockdown again. Our interview study coincides with this second wave, a time of highly differentiated circumstances between rural communities with zero COVID and a closed down capital, marked by pandemic fatigue and waiting for vaccines.

We recruited 12 informants for in-depth interviews about pandemic news experiences, building primarily on a broader sample from a qualitative questionnaire study conducted during the early lockdown period in spring 2020, in which 550 respondents had written about media use in early lockdown, and indicated if they were willing to be contacted again at a later date (Ytre-Arne and Moe, 2021). For the in-depth interviews we selected a small but diverse sample of informants in terms of gender, age, occupations and news habits, building on the information provided in the questionnaire replies. We ensured that the sample included people who had been differently affected by COVID with regards to their health, work, where they lived, or other personal circumstances. This two-step research design resembles a qualitative study conducted in the Netherlands (Broersma and Swart, 2021) and provides attention to how pandemic news experiences evolve over time.

The interview guide was developed following two main principles. First, we were interested in exploring each informants’ pandemic news media use in detail, and therefore asked a series of questions about what kind of media in general and news/journalistic content in particular our informants used in their daily lives. We explored how this had changed throughout the pandemic, and asked informants to think back to when they first heard of COVID and recount how they had experienced and interpreted news in different stages, in the context of their lives through the pandemic. Second, we were interested in understanding motivations behind any change in their media use. We expected that practical purposes, emotional states, and interest in maintaining public connection could be relevant here, and thus developed questions to investigate these dimensions. We also attempted to tap into people’s risk perceptions as part of their stories of what the pandemic had been like for them, and how they had reacted to different kinds of news.

The interviews were conducted on a digital platform, lasting for an hour or more, and transcribed in full afterwards. A clear impression already at the interview stage was that preoccupation with metrics was an obvious pattern in almost all the interviews, with references to numbers coming up repeatedly and in different contexts through the interview conversations. This concerned the local, daily infection rate in particular, and metrical pandemic information more generally. To delve deeper into what this interest in numbers could mean, we therefore decided to analyse the interviews focusing on this theme, inductively searching for recurrent patterns in experiences, rationalisations and reflections about pandemic metrics, across the transcripts from the different informants. As we have a small sample, we underline that general interest in infection rates, as well as the more specific interpretations we comment on, build on expressions shared between multiple informants. All names for informants quoted are pseudonyms.

## Analysis: monitoring the infection rate

The question we ask is why monitoring of infection metrics appeared meaningful to news users, and what these experiences can tell us about everyday engagement with data and the role of journalism in the pandemic information environment. Our analysis starts by answering the first question, focusing on monitoring of infection rates as a potential *coping mechanism* in the face of the practical and existential uncertainty brought about by the pandemic. Next, we look closer at the specific role of journalism, as opposed to other potential providers of pandemic metrics, highlighting why people experienced the *informational quality of metrics in journalism* as valuable.

### From fuzzy fear to everyday actions: monitoring as coping mechanism

For our informants, the mood of the early pandemic was characterised by precarity and uncertainty. Many mentioned images of overcrowded hospitals in Italy as the trigger that made them understand that coronavirus was different from earlier health crises: the COVID-19 pandemic would come to affect them and their local communities personally. Some observed the rapid acceleration and change of tone in official communication up to the press conference on March 12th, where the Norwegian government announced a national lockdown, as a transformative media experience. Following these events, most of our informants expressed a strong need to balance between following the news closely to keep up with a pressing societal crisis, and taking breaks from excessive media use to conserve energy for managing daily life. Our findings correspond here with several studies on how news users balanced information needs and attempts to avoid overload, in an emotionally charged situation (eg. De Bruin et al., 2021; Vandenplas et al., 2021). Monitoring the infection rate was an important part of the new, intensified news media repertoire:

Oh yes, absolutely, so, from March 12th and to the end of May I guess [...] I used the media much more to an extreme degree. I researched a lot about corona, and then perhaps especially […] local infection rates in the area and in the county, and also, international sites like John Hopkins, to see how it is going, how it was going in the world. (Kåre, man in his 40s, disability pensioner).

Kåre monitored the infection rate to *understand* what the pandemic was, “how it was going” and what it meant, locally and in the world. His statement exemplifies how perceptions of acute and world-altering crisis leads to intense information-gathering. Other informants described news use in early lockdown as “being paralyzed” (Susanne, advisor) or “I started consuming, consuming, consuming. And then I became exhausted” (Fatima, professor). Such shifting modes of information-seeking have previously been observed in reactions to political turmoil (Moe et al., 2019) and applied in analysis of the pandemic (Groot Kormelink and Klein Gunnewiek, 2021), also regarding the monitoring of infection rates (Radinsky and Tabak, 2022). Furthermore, images from Italian hospitals were interpreted in tandem with a monitoring of the infection rate, triggering ambivalent emotions of both fear and fascinations, resembling how Gray (2020) discusses the data sublime, the feeling of awe when data and visuals operate together.

A pattern in our interviews was that informants explained that their understanding of the pandemic, building particularly on metrics, soon found *practical* applications. This corresponds to findings by Mannell and Meese (2022) on how informants who primarily wanted to avoid pandemic news still found the daily infection rate useful, to make decisions about for instance travelling. Our informants used the infection rate and the broader conception it conveyed to assess risks – not only regarding threats to their own or others’ health, but also concerning special arrangements for holidays, family gatherings and work events, and mundane everyday actions such as shopping for groceries or using public transportation. With comparatively light restrictions in many parts of Norway after the first wave, there was sometimes a measure of choice within current guidelines, as opposed to a full-on lockdown. If shops were open but one was encouraged to avoid crowds, a decision had to be made about whether a specific errand was a necessity worth the risk. If events could go ahead on a small scale with some restrictions, considerations had to be made about how to act. Einar, a cultural sector worker in his 30s, monitored the infection rate from the very beginning for professional reasons, to try to understand which possibilities remained open to his line of work:

What happened now is also very related to my job, so, I must pay attention all the time to changes in the infection rate and try to predict the direction things are heading to, trying to plan a little. Ehm, well, [arranging an] event or not, and how things will happen if we do.

Informants thus explained their monitoring of the infection rate as a means for gaining *understanding,* but also of having *control* of the situation to determine their own response. For multiple informants, this had clear practical purposes, as we will give further examples of below. There is a parallel here to Radinsky and Tabak’s (2022) discussion of how everyday data practices during the pandemic gave people agency. At the same time, we see a surprisingly strong parallel to how Masson and Van Es (2020) discuss the use of a weather forecast site as a means for everyday planning. This suggest that that data monitoring practices and the agency experienced through it might be relatively similar across diverse contexts. For our informants, monitoring the infection rate was especially prevalent as governmental health advice became differentiated, as compared to the first lockdown in the spring of 2020. This implied that interest in infection rates – a new habit picked up in the immersive news use of early lockdown – stuck with our informants even as other aspects of pandemic news use changed.

However, the control offered by the infection rate went beyond practical purposes. We would argue that it first and foremost should be seen as a strategy, enabled by news media monitoring, to cope with the fear and anxiety caused by the pandemic. This was indicated by our informants’ strong interest in numbers in order to understand and tackle the pandemic in its early phases, during a relatively hard lockdown when any practical application of this monitoring would be minimal. Furthermore, although few informants explicitly mentioned fear or other emotions, nor did they say they feared getting sick, their vaguely formulated need to understand “how things are going”, “the direction we are heading”, references prospection of an uncertain future. It has been argued that *fear of the uncertain* is one, if not even *the* fundamental form of fear (Carleton, 2016), and that strategies of gaining control and understanding over an unknown phenomenon, like the pandemic, thus should be seen as strategies of coping with fear. This was clear among informants who expressed various forms of distress when monitoring the infection rate. An example was Fatima, a university professor with an international background, who had family ties to countries more severely affected than Norway by COVID-19:

I had been following the situation, so I saw certain patterns. And for example, I thought “oh my God, here they are too slow at the moment”. If things follow the same pattern, the virus is already out of control, and they are not closing down society yet. So, in a way it is a form of defense because as much as I trust the authorities, and I think in Norway they have done very well, but still – you still want a safety net, you want to still … to reassure yourself that … to have a kind of your own second opinion. Like “Okay I trust them” … but still, if they aren’t taking action then I am going to action.

Fatima’s account clearly illustrates a sense of gaining personal control by monitoring the infection rate and predicting how the situation will develop in different countries with the same measures, or lack thereof. Her statement also shows how this process is laden with affect, as she balanced her trust in Norwegian authorities with increasing anxiety, feeling that someone had to act but also the weight of addressing that responsibility. Thus, monitoring the infection rate had practical value, but it was also a coping mechanism for handling troublesome emotions. It should of course be stressed that these two aspects go hand in hand, as emotions are key tools for understanding the world and engagement with data should thus be understood as both emotional and practical (Kennedy and Engebretsen, 2020).

This was also clear in the case of Michael, a student enrolled in a prestigious study program located abroad, who moved back to Norway during the pandemic. Like Fatima, making comparisons between Norway and other countries he had personal ties to was part of how he evaluated the pandemic, forming personal outlooks and predictions. Here, infection rates were key, as the metrical quality seemingly enabled comparisons across contexts and into the future. The pandemic restrictions had potentially high personal consequences for Michael, as it could halt his education and income, putting future plans on hold and sending him “back home” indefinitely instead. It was therefore important for him that the infection rate would stay low during the summer and fall 2020, when travel restrictions were based on the notion of “green” versus “red” countries, calculated based on infection rates and population figures. He talked about monitoring numbers to see if the country he studied in would stay green so that he could travel there and continue his studies:

**Michael:** I remember the rate started to get close to 20 around two weeks before departure [refers to case count applied in calculation of red areas]. And all expectations at the time indicated it would pass. I think Belgium was the only country that had tipped 20 a bit before, and then I started to get nervous if I actually could travel where I wanted to go, and continue [his studies]. It was of course also sad on a general basis when things started to get worse again. Eh, and... as I said, I felt the increased need to more frequently... quite frequently update, go in and look again, «has there been any changes in the number», “what are people writing about it”, “do you think it will go a lot higher or not”? But we were afraid that it would tip 20. We had no idea that it would tip more than thousand.

**Interviewer**: Right(...)When you felt the need to monitor closer, did you check multiple times a day?

**Michael**: Yes, I could check every hour [laughs]. Or, it depends on what one is doing, but yes.

The uncertain situation Michael experienced did, as he describes, elicit a quite intense monitoring practice, checking updates by the hour on different websites as well as journalistic and social media commentary on the developments. During this part of the interview, Michael became more emotional, demonstrated by a trembling voice and tears as well as laughter, after having presented a curious and positive attitude in the interview so far. It seemed clear that the situation had been difficult for him, and that monitoring the infection rate had been an important part of trying to handle his reactions to the lack of control he experienced.

It might thus be that for some informants, monitoring eventually became more of a stressor than a coping mechanism, and even the number itself or its visualization would solicit an emotional reaction, a finding similar to what Radinsky and Tabak (2022) discuss. Another example was Gudrun, a student and bartender in their 20s, who talked about the immediate responses triggered by changing metrics and visualizations:

**Gudrun**: And as soon as it would point downwards [the graphic illustrating the infection rate], I would be like “pheeeew”…

**Interviewer**: So you felt it would help, like… […] when it went downwards?

**Gudrun**: A little perhaps, I am still like that [...] if there is a green arrow downwards, I know right away “oh yes that is good”.

**Interviewer**: Exactly.

**Gudrun**: But again, if it is a red arrow upwards, then...

**Interviewer**: Yes.

**Gudrun**: Then I get stressed again, but…I have been trying not to think too much about it.

As this interchange indicates, monitoring the infection rate would get a reaction, but it would also provide them with immediate information through simple cues: Green is good, arrow pointing down is good, red is bad, arrow pointing up is bad. This is a typical illustration for how our informants viewed data visualizations of the infection rate as valuable: As a quick overview of the current situation, to which one could immediately react. Journalistic news media were not alone in providing such visualizations, but nevertheless seemed important to how informants preferred to find and monitor metrical information. We will now look more closely at how informants experienced the role of news outlets in providing information on the pandemic infection rate.

### Valuable facts: the role of news outlets in infection monitoring

**Interviewer:** [Speaking of the early lockdown phase] What kind of news were important for you to catch?

**Susanne:** Well, it was, all things, infection and death rates, totally and comparatively, with countries as well. I sat on that website, and refreshed it, and studied it all the time, and checked and organized, I was in control, it is so silly [laughs]

**Interviewer:** How did you feel about it, was it the numbers that made you feel in control or…

**Susanne:** It is really about understanding where you yourself fit in, and like, how, how things will be. An attempt to set a course […] If you are at sea, as I often am, you need to know the area, to read the map, you need to know where the reefs are, where the currents are, it is a bit like that. (Susanne, woman in her 40s, former communications advisor).

Our informants valued the kind of news that allowed them to do this kind of mapping and get an overview of the complexities of the pandemic. Susanne compares the infection rate to a sea map: The map gives an overview, communicated through simple symbols, of phenomena that are highly complex, and that must be navigated in order to arrive safely on the other side. Importantly, she speaks of how she in the early phase of lockdown did substantial information gathering herself, nearly taking on the role of amateur epidemiologist and data analyst, with constant updates on an unspecified Web site. As such individual in-depth information gathering appears difficult to most people over time, journalism fulfills a function of providing information to the approximately informed and occasionally monitorial citizens (Ytre-Arne and Moe, 2018).

Our informants talked of journalistic news as mixed with other kinds of pandemic information, for instance mentioning municipality websites or social media posts as similar to local news, but also spoke about how their everyday journalistic news media habits were important for them in order to monitor the pandemic. They expressed that news outlets were particularly important for continued and easily accessible monitoring of infection rates - what Radinsky and Tabak (2022) conceptualizes as the data practice of *scanning*. Scanning as a data practice is often connected to media routines where one checks certain websites, as well as to the technological affordances smartphone apps provide for quick and easy updates. For our informants, it seemed like force of habit often decided which outlets were used to monitor the infection rate: Karen, a civil servant and mother, stressed how three specific news outlets had followed her since her university days, Kåre, a disability pensioner, emphasized how he on principle trusted the free sources VG and NRK to provide him with important societal information already before the pandemic, while Gudrun, a student, mentioned push alerts from the VG app providing live updates throughout the day.

That journalistic outlets became an important source of information was thus partly due to habit, convenience and availability. However, the way in which they provided information was also important. Especially visualizations would influence the informants’ choices of preferred news source, so that they looked to news outlets that offered understandable and prominently placed visualizations. Several said that they preferred to check the online version of Norway’s largest tabloid paper, VG, every day, even if this was not their go-to news source pre-pandemic. While VG is part of a tabloid tradition, the newspaper has a solid position in areas such as news and political reporting, and a long-standing tradition for service journalism (Eide and Knight, 1999). With these possible precursors, the attraction of VG in the pandemic crisis was, as mentioned in our introduction, a perception that this newspaper offered the best infographics. VG had a large and interactive statistics bank with graphs detailing development over time, and maps indicating the infection rates in different areas, including not only on national level but also encompassing both Norwegian municipalities and details from neighboring countries. This adaptability was stressed by multiple informants as important to plan their pandemic life – for example by Tom, a freelancer with a chronic disease that made him more vulnerable for serious illness, who used it to plan whether he would visit a certain part of Sweden or not. Furthermore, VG had a clearly visible banner on the newsfront which included the already mentioned red or green arrow, to indicate whether the infection rate was increasing or decreasing. This would give an immediate overview of the “status of the day” during our informants’ everyday media routine. As Kennedy and Hill (2018, p. 844) argue, “[d]ata come into being through visual design”, and maps that clearly divided Norway and the world in “green” and “red” area; as well as arrows interpreting the pandemic as going “up” or “down”, were clearly perceived by our informants as data that could provide agency in the everyday (Radinsky and Tabak, 2022) – a parallel to the mundaneness of the weather forecast (Sturken, 2001) and especially how data visualization makes it easy to monitor rainfall (Masson and Van Es, 2020).

A clear visualisation of the infection rate was seen as a useful tool for mapping the situation, for gaining a sense of control, and, we would argue, as valuable pandemic journalism in the form of information. What might lie behind this is a belief that numbers cannot lie – they were experienced as hard evidence. This points towards an interesting relationship between how numbers are trusted on the basis of being objective, and how they can elicit emotion (Gray, 2020; Kennedy and Hilll, 2018, Radinsky and Tabak, 2022). It is worth noting that this hard evidence-quality of numbers was explicitly stated by some informants who normally avoided news and mainstream journalistic media, for different reasons. Fatima expressed how she, also pre-pandemic, found news emotionally distressing. Furthermore, because the Norwegian political landscape was unfamiliar to her, having moved to Norway as an adult, she was not interested in knowing more about it. However, she soon started to follow the public broadcaster NRK and a specific newspaper in her birth country in order to monitor the infection rate when the pandemic spread. She told us that she followed the numbers because they *tell a story,* and this story was – partly – about how serious the pandemic was in different parts of the world. In other words, Fatima treated numbers as *indicative* measures of the severity of the pandemic, and the infection rate as journalistic content was her main motivation to return to news use, and to specific news outlets. At the same time, these specific news outlets were chosen because she already trusted them – either because of previous habit and experience or because it was a public broadcaster. Trust in numbers was thus not only based on their ‘inherent’ objectivity, but closely intertwined with existing trust relationships with news sources, as already described in the literature (Kennedy and Hill, 2018; Radinsky and Tabak, 2022). As valuable journalism, metrics is thus part of existing trust relationships with the media.

A quite different story was told by Erik, an insurance adviser in his 30s who expressed scepticism towards mainstream news media. He did not believe that media provided “fake news”, but argued that all news media would have an ideological angle in their coverage as well as ideological reasons for publishing exactly the content that they did. He also frequently observed what he called “fear-mongering” from the media. However, he regarded Covid infection rates as an exception from these criticisms:

But it is, it is the reality, I, even as sceptical as I am towards the media I trust that it is correct what they say regarding infection rates and regarding number of deaths and all that stuff, […] cause here we are, in an unusual situation, with a whole new thing, I just think it is nice that the media machinery actually focuses on this.

As we can see, numbers are seen as facts (Porter, 1996), and therefore as objective indicators of how bad – or well – it is going with the pandemic. This is in line with Billig (2021) who argues that numbers are treated in a “magical” way in the rhetoric of the British government, but in our case the magic works bottom-up: numbers, believed to be “pure facts”, become valuable journalistic content because people use them to make sense of the pandemic and assess risk. It should be noted that Erik, as well as some other informants, spoke about the photos from Italy and China in the same vein: clear evidence of the severity of the pandemic. Interestingly, numbers and images, often seen as opposites when it comes to informational and emotional potential, held some similar functions for our informants: they were interpreted as undisputable proof telling a story laden with emotion, and perhaps even worked together to create a feeling of the sublime (Gray, 2020). Objectivity and affect could together be used in sense-making, which were key for why metrics were experienced as engaging (Kennedy and Engebretsen, 2020) and thus as valuable information.

## Conclusion

We have analysed news users’ experiences with monitoring pandemic infection rates, as an expression of a perception of what constitutes valuable journalism in an ongoing societal crisis, drawing on a qualitative interview study conducted in Norway. Our study contributes to the growing literature on pandemic news use, with a specific focus on one central aspect of pandemic news experiences, providing a qualitative user perspective on interpretations of metrical information as part of the pandemic information landscape.

The monitoring of infection rate was a clear pattern in the news experiences of our informants during the first nine months of the pandemic, that is, to the point of the interviews. We argue that monitoring of infection rates should be understood as a means to *understand* the pandemic, as a complex societal phenomenon with practical and existential implications. Initially, many informants monitored metrics to make sense of the scope and the severity of the pandemic, but soon monitoring developed into a practical information shortcut as well as an emotional coping mechanism. The infection rate would be monitored for personal and professional reasons, assessing whether a trip or family visit would be a health issue, or how one could best make plans at work. At the same time, monitoring the infection rate had an affective function for many informants. Departing from theories of fear of the unknown as a primordial form of fear, we argue that the intense monitoring of the infection rate should be seen as a coping mechanism transforming fuzzy fear into a feeling of control. However, the intense monitoring served the opposite purpose for some of our informants, being a stressor when the infection rate increased, rather than a means of emotional coping.

Regular and frequent updates on the infection rate, as well as effective and striking visualisations, was the journalistic content most sought after by our informants. Visualisations were experienced as useful because they were easy to process, and numbers were understood as telling stories of seemingly indisputable facts, that nevertheless were emotionally charged. Hence, reporting of numbers was seen as a valuable form of journalism also for people who normally would avoid legacy news media for different reasons. We thus find that there was considerable informational value and emotional salience ascribed to pandemic metrics, seemingly bypassing other criticisms and challenges to the relevance of journalism.

As indicated above, there are questionable aspects in the interpretations of numbers as factual information telling a particularly truthful story of the pandemic. It is also important to note, in this context, the relatively privileged position of our informants in a country in which the infection rate stayed comparatively low, and therefore could offer comfort through long stretches of the COVID crisis. What we find most interesting, however, is how people integrated scanning of metrics into monitorial practices to make sense of a world in crisis, looking for very specific bits of information that they felt could tell them something true and important of a complex big picture, while helping them navigate in daily life. Infection metrics were essential to the reporting of the major international news story of the time, with considerable societal implications, but also a form of service journalism assisting in daily decisions. The infection rate seems to have taken on the status of a weather forecast for how to navigate the stormy seasons of the pandemic. This poses the question of how journalism could or should provide similar information in other types of societal crises, such as war and conflict, persistent social inequalities, or climate change.
